# Statistical and Diagnostic Properties of pRRx Parameters in Atrial Fibrillation Detection

**DOI:** 10.3390/jcm11195702

**Published:** 2022-09-27

**Authors:** Szymon Buś, Konrad Jędrzejewski, Przemysław Guzik

**Affiliations:** 1 Institute of Electronic Systems, Faculty of Electronics and Information Technology, Warsaw University of Technology, Nowowiejska 15/19, 00-665 Warsaw, Poland; 2 Department of Cardiology-Intensive Therapy and Internal Disease, Poznan University of Medical Sciences, 60-355 Poznan, Poland

**Keywords:** atrial fibrillation detection, cardiac arrhythmia, electrocardiography, heart rate variability

## Abstract

Background: We studied the diagnostic properties of the percentage of successive RR intervals differing by at least x ms (pRRx) as functions of the threshold value x in a range of 7 to 195 ms for the differentiation of atrial fibrillation (AF) from sinus rhythm (SR). Methods: RR intervals were measured in 60-s electrocardiogram (ECG) segments with either AF (32,141 segments) or SR (32,769 segments) from the publicly available Physionet Long-Term Atrial Fibrillation Database (LTAFDB). For validation, we have used ECGs from the Massachusetts Institute of Technology–Beth Israel Hospital (MIT–BIH) Atrial Fibrillation Database. The pRRx distributions in AF and SR in relation to x were studied by histograms, along with the mutual association by the nonparametric Spearman correlations for all pairs of pRRx, and separately for AF or SR. The optimal cutoff values for all pRRx were determined using the receiver operator curve characteristic. A nonparametric bootstrap with 5000 samples was used to calculate a 95% confidence interval for several classification metrics. Results: The distributions of pRRx for x in the 7–195 ms range are significantly different in AF than in SR. The sensitivity, specificity, accuracy, and diagnostic odds ratios differ for pRRx, with the highest values for x = 31 ms (pRR31) rather than x = 50 (pRR50), which is most commonly applied in studies on heart rate variability. For the optimal cutoff of pRR31 (68.79%), the sensitivity is 90.42%, specificity 95.37%, and the diagnostic odds ratio is 194.11. Validation with the ECGs from the MIT–BIH Atrial Fibrillation Database confirmed our findings. Conclusions: We demonstrate that the diagnostic properties of pRRx depend on x, and pRR31 outperforms pRR50, at least for ECGs of 60-s duration.

## 1. Introduction

Atrial fibrillation (AF) is a tachyarrhythmia with uncoordinated atrial electrical activation and ineffective atrial contraction [1,2,3]. Diagnosis of AF is based on an electrocardiogram (ECG) with irregular RR intervals (the distances between the peaks of R waves of the QRS complexes reflecting the electrical depolarization of ventricles and measuring the duration of each cardiac cycle), the absence of distinct repeating P waves, and irregular atrial activation. AF must be documented in an entire 12-lead ECG of a duration of at least 8–10-s. If a single ECG strip is used, an arrhythmic episode is considered AF if it lasts at least 30 s [1,2].

AF is the most common sustained cardiac arrhythmia, and it is the only available cardiac rhythm for many people. Heart palpitations, symptoms of irregular pulse, worsened exercise tolerance, dyspnea, and sometimes angina can accompany AF. However, many individuals are asymptomatic or experience mild or unspecific symptoms easily related to advancing age, emotions or effort, or other diseases such as hyperthyroidism [4]. Consequently, AF may be undetected or is found accidentally during a routine check-up or a medical visit for another reason [1,2].

AF increases the risk of excessive morbidity with debilitating clinical consequences and premature death. This arrhythmia always increases the risk of severe arterial thromboembolism, resulting, for example, in an ischaemic stroke of the brain. Therefore, AF must be appropriately diagnosed and actively screened for, particularly in high-risk populations such as older age and patients with hypertension, diabetes, heart failure, or valvular disease [5,6].

Many systems used for AF screening can record ECGs of various lengths, ranging from a couple-of-second 12-lead ECG to recordings of several weeks with the long-term Holter ECGs or ECG bio-patches attached to the skin. Implantable loop recorders are an invasive option for even more prolonged ECG monitoring lasting up to two years.

Visual inspection and ECG analysis are the gold standards for AF diagnosis. With technological advancement, the amount of data with long-term ECGs gradually increases, so a quick analysis of such recordings becomes challenging. New approaches to automatic or semiautomatic analysis are proposed, many of them based on heart rate variability (HRV) analysis [7]. HRV uses mathematical analysis of RR interval time series. RR interval is the cardiac cycle duration measured between two consecutive QRS complexes in a continuous ECG. HRV was developed many years ago. Its primary use was limited only to RR intervals of sinus rhythm (SR) origin for either physiological analysis or prediction of mortality in different diseases.

Due to different distributions of HRV-derived parameters in AF and SR [8], HRV has gained new interest in AF detection in ECG [9,10,11], as well as from wearable devices [12,13]. Several authors used feature selection methods to find the most relevant HRV parameters for AF detection [14,15]. Others incorporated HRV to predict future occurrence of AF [16,17].

The percentage of successive RR intervals differing by at least 50 ms (pRR50) is a particular form of pRRx parameter. Ewing et al. [18] proposed the total number of successive RR intervals that differ by at least x ms (RRx count) for monitoring cardiac parasympathetic activity. They analyzed two threshold values: x = 50 ms and x = 6.25% of the previous RR interval. The computationally simpler 50 ms threshold was later widely adopted, and Bigger et al. [19] proposed a relative statistic pNN50 (percentage of normal-to-normal RR interval differences over 50 ms). The “NN” in the name emphasizes that the analyzed R-waves are of sinus origin [1,19]. However, the same mathematical analysis can be conducted for different cardiac rhythms, including AF. RR intervals are not normal (i.e., of sinus origin) in AF and thus should always be labeled as RR and not as NN.

So far, the threshold values x other than 50 ms have rarely been analyzed. Mietus et al. [20] used pRRx with x ranging from 4 ms to 100 ms to compare various groups such as healthy people versus patients with heart failure, sleeping vs. awake states, or young vs. elderly subjects. In all cases, thresholds < 50 ms allowed for better discrimination between the studied groups. Torres et al. [21] applied pRRx with x from 10 ms to 50 ms to distinguish healthy subjects from survivors of acute myocardial infarction, and thresholds < 50 ms performed better. Saiz-Vivo et al. [22,23] used HRV indices, including pRR20 and pRR50, to analyze 500 beats preceding onsets of AF and distinguish between healthy and AF subjects. In several studies [24,25,26,27], various pRRx indices with x from 5 ms to 500 ms were used to distinguish between four cardiac rhythms (AF/SR/noisy/other) [28]. Jovic et al. [29] combined various HRV parameters with pRR5, pRR10, pRR20, and pRR50 to differentiate between nine cardiac rhythms, including AF [30].

We have recently analyzed the diagnostic properties of several HRV parameters, including the mean of RR intervals (mean RR); the standard deviation of RR intervals (SDRR); the standard deviation of points perpendicular to the line of identity (SD1) and along the line of identity (SD2) from the Poincare plot analysis; the power of low-frequency (LF: 0.04–0.15 Hz) and high-frequency (HF: 0.15–0.4 Hz); and pRR10, pRR30, pRR50, pRR70, and pRR90 [31]. Among these parameters, pRR30 had the highest area under the curve (AUC) [32]. Different combinations of the HRV parameters, used as input features for ML classifiers, showed that the sets with pRR30 outperformed other HRV feature sets, including those with pRR50. Conroy et al. [33] used parameters analogous to pRRx to detect AF in the photoplethysmographic signal (PPG). Instead of RR intervals, interbeat intervals (IBI) from PPG were used, and the highest AUC was yielded for x = 35 ms. Ramesh et al. [34] used HRV features, including pRR20, RR20, pRR50, and RR50 (and analogous parameters from PPG), for AF detection in ECG and PPG.

Altogether, these data strongly suggest that various x threshold values for the difference between two successive RR intervals might be more helpful in detecting AF. However, the 50 ms threshold (pRR50) has been used for many years as a commonly accepted practice in HRV analysis.

The primary aim of this study was to systematically explore the diagnostic properties of the pRRx as a function of the different x thresholds for differentiating AF from SR in 60-s ECG segments. The secondary aim was to compare the diagnostic properties of the pRR50 with the pRRx found to have the optimal diagnostic properties for AF detection in the same 60-s ECG segments. This part of the study is presented separately in Appendix A.

## 2. Materials and Methods

### 2.1. Data

For this study, we used anonymized data from the Long-Term Atrial Fibrillation Database (LTAFDB) [35,36]. It contains 84 long 24-h Holter electrocardiographic (ECG) recordings sampled at a rate of 128 Hz, the information about locations of R-waves, and the type of corresponding cardiac beats (normal, supraventricular, ventricular, atrial fibrillation, and technical artifact). The LTAFDB database contains ECG recordings from patients with paroxysmal AF and other arrhythmias. We selected only uninterrupted ECG fragments with either AF or SR of at least 60-s duration for further analysis. We discarded the segments labelled as different rhythms.

**Figure 1:**
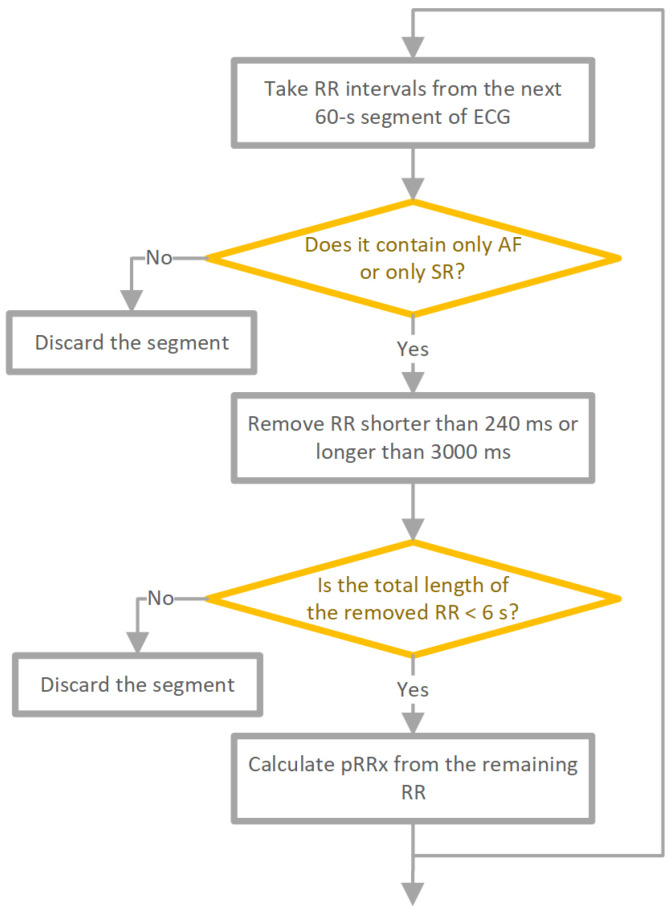
Data preprocessing scheme. RR—interval between peaks of consecutive R-waves; ECG—electrocardiogram; AF—atrial fibrillation; SR—sinus rhythm; and pRRx—percentage of successive RR intervals differing by at least x ms.

The data-preprocessing scheme is presented in Figure 1. The RR interval time series were cut into 60-s separate, neighboring segments. For a segment to be labeled SR, each RR interval had to be of SR origin. If it was not, for example, it was a atrial or ventricular beat, the segment was removed from further analysis. For AF segments, each cardiac beat needed to be AF, and if a ventricular beat was found, such a segment was also excluded.

For SR and AF, to limit the number of potentially unidentified technical artifacts, RR intervals shorter than 240 ms or longer than 3000 ms were removed. Additionally, RR intervals corresponding to ventricular premature beats were also removed from both SR and AF ECGs. For SR only, premature supraventricular beats were removed too. Segments with a total length of excluded RR intervals exceeding 6 s were also discarded from the analysis. The total number of 60-s RR series after preprocessing was 64,910 (32,141 AF, 32,769 SR).

As the sampling rate of 128 Hz corresponds to the precision of 7.8125 ms, we quantized pRRx thresholds x into 7.8125 ms bins. The absolute values of the differences between consecutive RR intervals were measured and used to calculate pRRx for the x ranging from 7.8125 to 195.3125 ms in 7.8125 ms steps for both SR and AF 60-s segments. To improve the paper’s readability, we use only the integer part of x in ms in the names of parameters, e.g., pRR7 instead of pRR7.8125.

### 2.2. Software Tools

We used Python programming language (version 3.9, Python Software Foundation, Wilmington, DE, USA) for all the analyses.

### 2.3. Statistical Analysis

First, we analyzed the distributions of pRRx obtained for different x values separately for SR and AF using histograms. Based on the histograms and Shapiro–Wilk test results, we concluded that pRRx do not have normal distributions either in AF or in SR. Consequently, we used a percentile scale to describe the distributions and mostly applied nonparametric statistical techniques for data analysis.

Next, using the Wilcoxon test [37], we made paired comparisons for pRRx with different x (separately for SR and AF). We conducted the unpaired analysis comparing pRRx for the same x between SR and AF using the Mann–Whitney test [38]. The associations between pRRx values for different x were analyzed with the Spearman correlation [39] and presented as heatmaps with rho correlation coefficients. To analyze the differences between two pRRx with different x, we calculated the mean difference for quantifying bias, and the standard deviation (SD) of the differences for each pair of pRRx. As the data distribution was not normal, we defined the limits of agreement (LoA) as the range between the 2.5th and 97.5th percentile of the distribution of the differences. To analyze the diagnostic properties of pRRx with different x, the area under the curve (AUC) from the receiver operator curve (ROC) characteristics was calculated [32]. We identified the optimal cutoff values for each x using Youden’s Index [40], which maximizes the sum of sensitivity and specificity of AF/SR differentiation.

For each threshold x with the optimal cutoff value for differentiating between AF and SR, we calculated several classification metrics [41], namely, accuracy, specificity, sensitivity, F1-score [42], positive predictive value (PPV), negative predictive value (NPV), and diagnostic odds ratio (DOR) [43]. For the estimation of classification metrics’ 95% confidence interval (CI), we used a nonparametric bootstrap with 5000 samples [44]. We analyzed all measures as functions of the threshold value x of pRRx in the range of 7 to 195 ms.

As the use of the pRR50 parameter is a de facto standard, we decided to compare its diagnostic properties against the optimal pRRx for AF detection. The optimal cutoff values for both parameters were estimated with the Youden criterion [40], and we used a nonparametric bootstrap to compare the distributions of the classification metrics with optimal cutoffs to verify whether the diagnostic properties of different thresholds are not random. For the sampling frequency of 128 Hz in the LTAFDB, the threshold x = 54 ms is equivalent to x = 50 ms, so pRR54 was used as an equivalent of pRR50.

## 3. Results

### 3.1. Data Distribution Analysis

**Figure 2:**
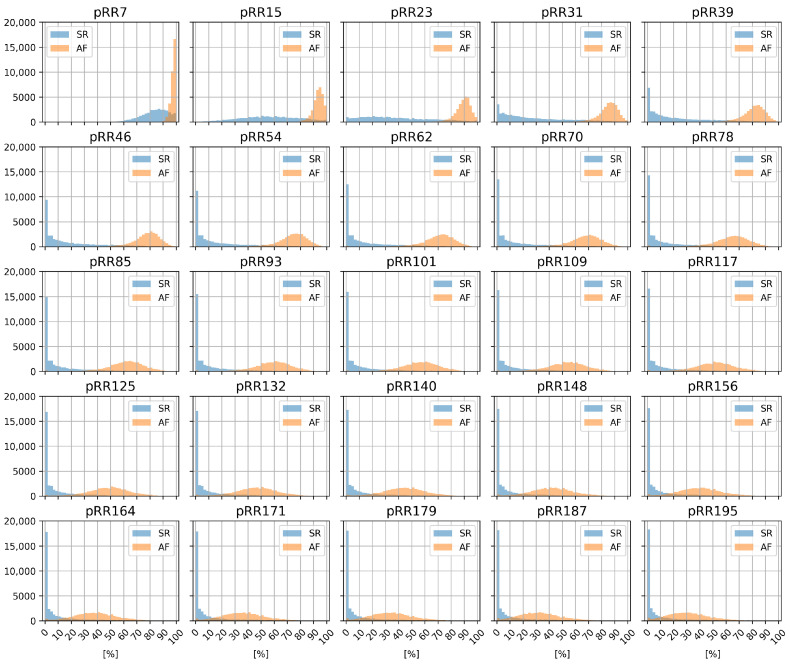
Histograms of percentages of successive RR intervals differing by at least x ms (pRRx) for sinus rhythm (SR) and atrial fibrillation (AF) for different x thresholds.

Figure 2 shows pRRx histograms for SR and AF for different x threshold values between 7 and 195 ms. The histograms for SR and AF partially overlap—for the same x, the left part of the SR distribution covers the right part of the AF distribution. The overlap is notable for very low (pRR7, pRR15) and very high values of the threshold x (pRR101–pRR195), but the distributions are better separated between these extremes.

**Figure 3:**
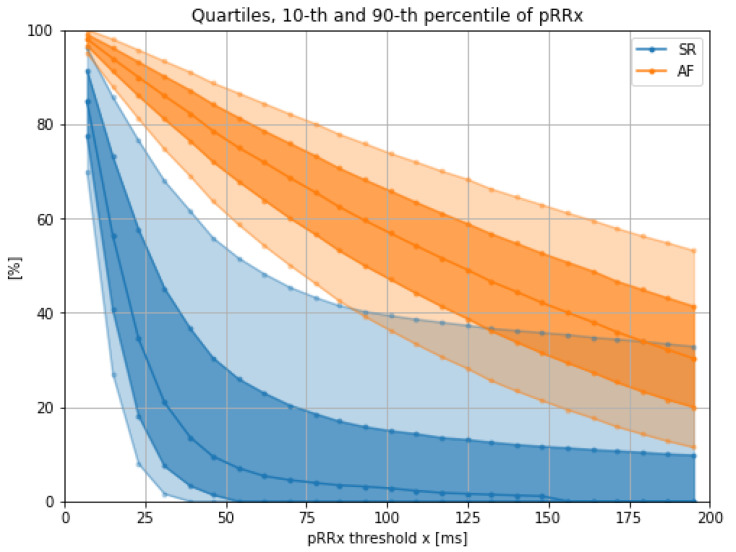
Medians (bolder line), interquartile ranges (darker band), and 10th to 90th percentile ranges (lighter band) of the percentages of successive RR intervals differing by at least x ms (pRRx) for sinus rhythm (SR) and atrial fibrillation (AF) for different x thresholds.

Figure 3 presents medians (bolder line), the 25th to 75th percentile range (darker band), and the 10th to 90th percentile range (lighter band) for AF (orange) and SR (blue). Median values of SR and AF never cross or overlap in the whole range of studied x. Interestingly, there is also no overlap between the 75th percentiles of pRRx for SR and the 25th percentiles of pRRx for AF. Additionally, the 90th percentile of pRRx for SR does not cross with the 10th percentile for AF in the range of x from 15 to 85 ms. It suggests that the optimal value of threshold x for differentiating between AF and SR lies within this range.

### 3.2. Correlation

**Figure 4:**
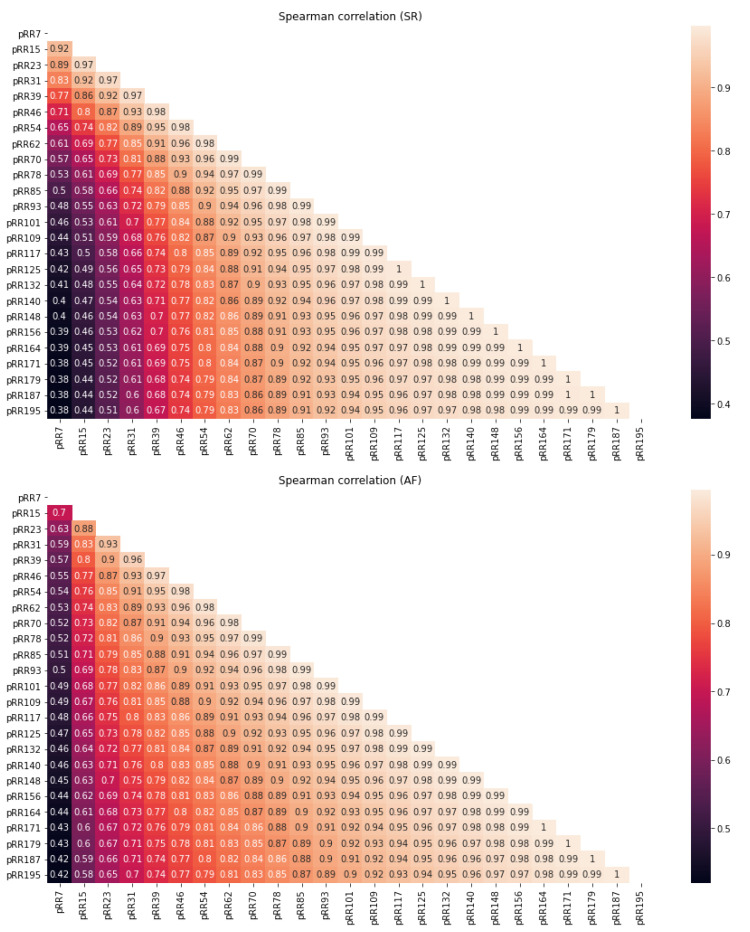
Heatmaps of Spearman correlation with rho coefficients between the percentages of successive RR intervals differing by at least x ms (pRRx parameters) for different x thresholds for sinus rhythm (SR, **top panel**) and atrial fibrillation (AF, **bottom panel**) segments of 60-s electrocardiograms.

Figure 4 shows two heatmaps with rho coefficients of Spearman correlations between pRRx for the different x thresholds in SR (the upper panel) and AF (the lower panel). For most x thresholds, particularly for lower values, the correlation coefficients are higher for SR than for AF. For the highest values of x, these correlations are above 0.95, both for SR and AF.

The rho coefficients decrease for SR and AF as the distance between two pRRx increases. Interestingly, this effect is more pronounced for lower x. For instance, in AF recordings, the rho coefficient drops from 0.7 for the pair (pRR7, pRR15) to 0.63 for (pRR7, pRR23). In contrast, rho is 1 for two pairs (pRR195, pRR187) and (pRR195, pRR179).

In AF, the pRRx with the lowest x (pRR7) has the strongest correlation with pRR15 (0.7), which is lower than in SR (0.92). However, the weakest correlation (between pRR7 and pRR195) is higher in AF (0.42) than in SR (0.38). It shows that for the lower values of x, the range of correlations is much wider for SR than for AF. Additionally, it suggests that the strength of correlations between various pRRx changes non-linearly for SR and AF.

### 3.3. Difference Analysis

**Figure 5:**
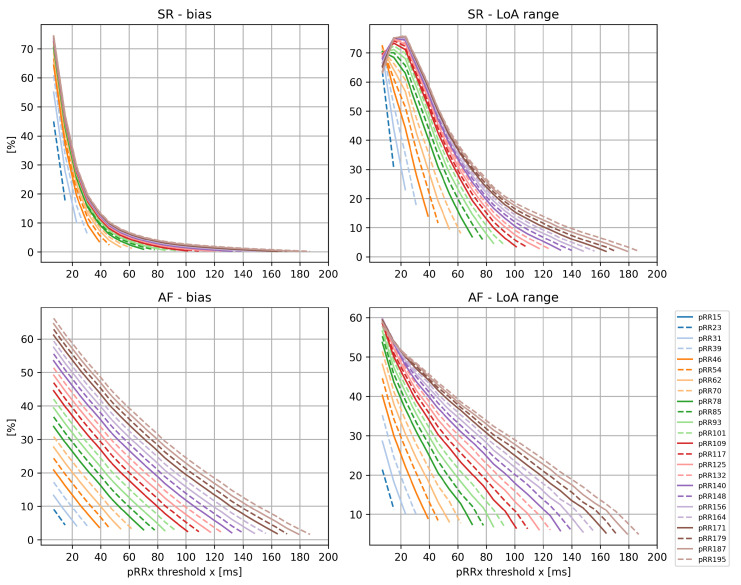
Summary of the analysis of the differences between the pairs of the percentages of successive RR intervals differing by at least x ms (pRRx parameters). Mean difference (bias) and 95% limits of agreement (LoA) range in sinus rhythm (SR, **top**) and atrial fibrillation (AF, **bottom**).

Figure 5 separately summarizes the differences between all pairs of pRRx parameters for AF and SR. For each pair of pRRx parameters, e.g., pRR7 and pRR46, the mean difference (bias) and LoA (the range between 2.5th and 97.5th percentile of the differences’ distribution) were computed.

There are visible differences in the relations of biases or LoAs of the differences for all possible pairs of pRRx with various x values. The bias distributions for different x resemble one of the reciprocal functions and are non-linear for SR and nearly linear with a negative slope for AF. In SR, for the x thresholds starting from 78 ms onward, the LoA values of the differences between different pairs of pRRx initially increase to their peak and then decline reciprocally. No similar early increase in such LoA is present for AF, in which these lines nearly linearly decrease from the maximal values for the closest to minimal values for the most distant pRRx pairs.

### 3.4. Area under ROC Curve (AUC)

**Figure 6:**
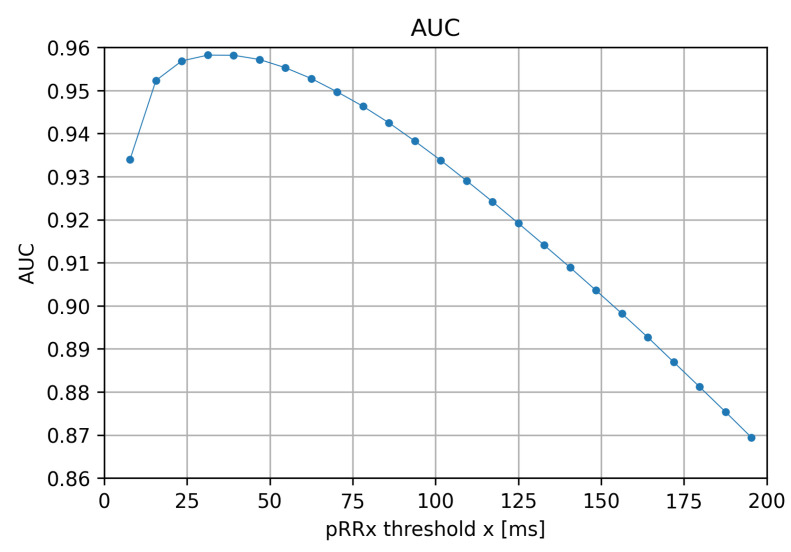
Area under curve (AUC) of the percentages of successive RR intervals differing by at least x ms (pRRx parameters) for the differentiation of AF from SR as a function of the x threshold.

The AUCs for the differentiation of AF from SR, shown in Figure 6, have their peak values exceeding 0.94 for the range of x from 15 ms to 85 ms (identical with the no overlapping zone between the 90th percentile of the pRRx distribution of SR and 10th percentile for AF as visible in Figure 3). AUCs gradually decrease to 0.87 for the highest x thresholds. In other words, pRRx for lower x values has a much better AUC for distinguishing the SR from AF recordings. The maximal AUC value can be one of the criteria for selecting the optimal x threshold for differentiating SR from AF with pRRx. In our study, the maximal AUC value (0.958) was for x = 31 ms, i.e., pRR31.

### 3.5. Determining Optimal Cutoff Values for Different pRRx

**Figure 7:**
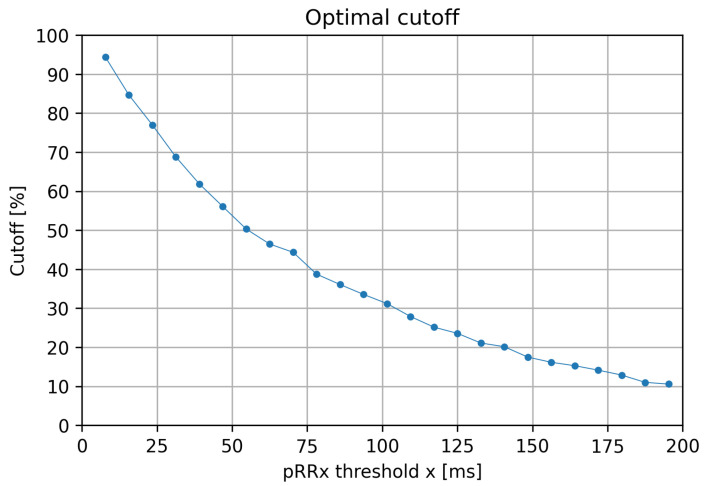
Optimal cutoff values based on Youden Index the percentages of successive RR intervals differing by at least x ms (pRRx parameters) as a function of the x threshold.

Using Youden’s index, we selected optimal cutoff values for all pRRx (Figure 7). Notably, the cutoff values for pRRx strongly and negatively depended on their x threshold.

**Figure 8:**
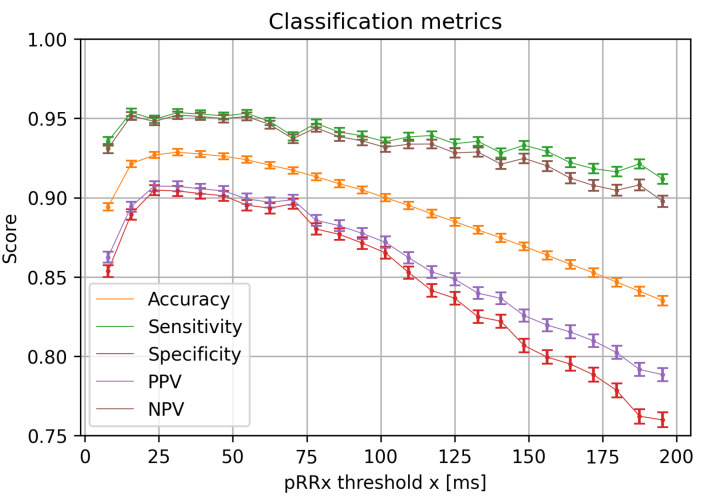
Medians and 95% confidence intervals of diagnostic classification metrics for optimum cutoff values for the percentages of successive RR intervals differing by at least x ms (pRRx parameters) as a function of the x threshold. PPV—positive predictive value, NPV—negative predictive value.

Next, the nonparametric bootstrap estimated the statistical metrics of AF detection using pRRx parameters with the optimal cutoffs. Figure 8 shows the median values and 95% CI of the classification metrics for optimal cutoffs of pRRx in relation to the x threshold. Although there is a gradual slow decline in the sensitivity of pRRx, its median exceeds 0.9 in the whole studied range of x. For the NPV, this relation is similar, with the lowest values above 0.89 for the largest x = 195 ms. Gradual decline with the increasing x values is also visible for both PPV and specificity. However, the decline is more rapid and deeper, from 0.89 to 0.77 (PPV), and from 0.88 to 0.72 (specificity) for x thresholds above 50 ms.

### 3.6. The Diagnostic Odds Ratios for Optimal Cutoffs for Different pRRx

**Figure 9:**
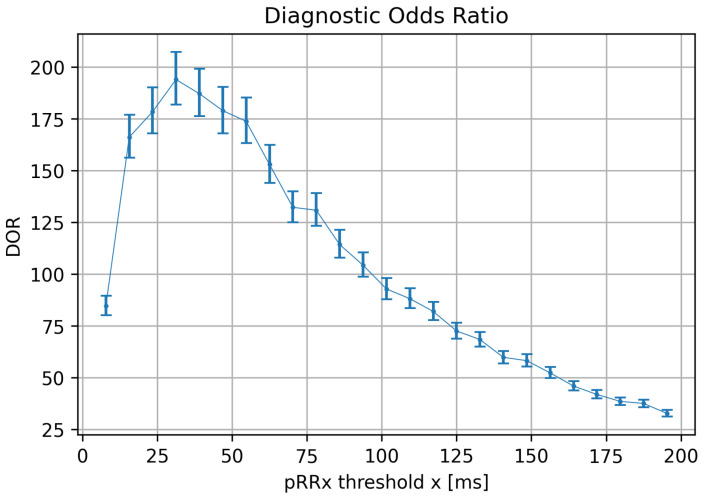
Medians and 95% confidence intervals of diagnostic odds ratio (DOR) for optimum cutoff values for the percentages of successive RR intervals differing by at least x ms (pRRx parameters) as a function of the x threshold.

Figure 9 presents median values of DOR with 95% CI for optimal cutoffs for different pRRx in relation to the x value. The highest DOR of 194.01 is observed for pRR31. In other words, the odds of pRR31 being greater or equal to the cutoff = 68.79% (Figure 7) is nearly 200 higher for the AF presence than in its absence. The next highest DORs are for pRR39, pRR46, and pRR25, all exceeding 175. DORs for pRRx for x up to 81 ms are at least 125, then gradually decline with increasing x values, reaching a minimum of 32.68 for x = 195 ms.

### 3.7. Comparison of pRR50 and pRR31

**Figure 10:**
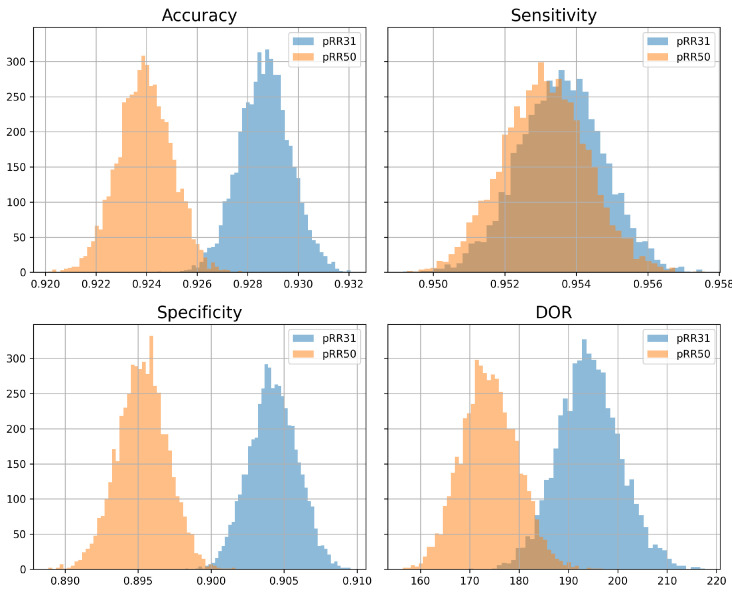
Histograms of classification metrics for optimum cutoff values for pRR31 and pRR50 (the percentages of successive RR intervals differing by at least 31 ms and 50 ms, respectively). DOR—diagnostic odds ratio.

The threshold x = 50 ms of pRRx is most broadly used and analyzed in HRV literature, including HRV-based AF detection methods. On the other hand, in our study, pRR31 achieved the highest accuracy (Figure 8) and DOR (Figure 9). We used a nonparametric bootstrap to compare the distributions of classification metrics obtained by pRR31 and pRR50 with optimal cutoffs to verify whether better diagnostic properties of different thresholds are not random. For the sampling frequency of 128 Hz in the LTAFDB, the threshold x = 54 ms is equivalent to x = 50 ms, so pRR54 was used as an equivalent of pRR50. Figure 10 shows the histograms of accuracy, sensitivity, specificity, and DOR for pRR50 and pRR31. All the metrics are visibly higher for pRR31 than for pRR50, except for sensitivity, which is only slightly higher for pRR31. The Shapiro–Wilk test verified that accuracy, sensitivity, and specificity have normal distributions and DOR does not. The histograms and the results of paired *t*-test (accuracy, sensitivity, and specificity) and Wilcoxon test (DOR) demonstrate that better performance of pRR31 is not random.

## 4. Discussion

In this study, we summarize various statistical properties of pRRx as functions of the x threshold both for AF and SR. All possible pairs of pRRx, regardless of the x distance separating them, are well correlated (Figure 4). Good correlations with rho at least 0.7 were present for all pRRx starting from x = 46 ms and most for x = 39 ms in SR recordings. In AF, all pairs of pRRx are well correlated, starting from x = 31 ms and then for most x = 23 ms. For higher x, pairs of pRRx are perfectly correlated. Lower pRRx values provide more information than higher pRRx. For instance, compared to pRR100, the pRR31 informs about the percentage of neighboring RR intervals, which differ not only from 100 ms but also in the lower range between 31 and 100 ms. In other words, using pRRx with higher x thresholds is more aggressive as it filters out much important information in the lower x ranges. Another issue is that good correlation does not mean that all pRRx parameters are the same and thus replaceable. Besides, caution should be exercised for the lowest x = 7 ms, for which the rho values drop to 0.38 and 0.42 for SR and AF, respectively. This analysis shows that various pRRx provide different information, particularly those with lower x.

The analysis of differences between different pRRx (Figure 5) reveals that their mean difference (bias) and the 95% limits of agreement range have distinct properties in AF and in SR. It shows that the decline of pRRx differences is more dynamic for SR than AF, even for the neighboring x. This analysis also demonstrates that pRRx with different x, even for the closest pairs of pRRx, do not provide identical information.

The optimal cutoffs for pRRx depended differently on the threshold x in a wide range from 7 ms to 195 ms. The best diagnostic features for AF detection are for the x range between 15 and 85 ms. It was repeatedly indicated by comparing pRRx 90th percentile for SR and 10th percentile for AF (Figure 3), then AUC over 0.94 (Figure 6), and finally with odds ratios exceeding 110 for specific cutoffs (Figure 9). Out of several possible x values within this range, x = 31 ms appears to be the best.

pRR50 is the most broadly studied pRRx parameter in physiological and clinical studies with ECG with sinus rhythm and for the differentiation of AF from SR [45,46,47,48]. Only a few studies explored pRRx with threshold values x different than 50 ms in AF detection [22,23,25,26,27,29,31,33]. Among them, only one study contained an analysis of the distribution of pRRx in AF and SR [26], and two had AUCs of the parameters [31,33]. However, neither analysis was in-depth.

Our study is more detailed, systematic, and focused on the whole pRRx family in a broad range of x thresholds. It is probably the first analysis investigating various statistical properties of pRRx exclusively as a function of x for the differentiation of AF from SR.

This study provides practical consequences for distinguishing AF from SR in 60-s ECG segments. First, although pRR50 is within the range of x values with very good diagnostic properties, it is not the best. Second, for the 60-s ECG segments, the x = 31 ms outperforms other pRRx (including pRR50), particularly when comparing DORs. The odds of pRR31 exceeding 68.79% (Figure 9) is nearly 200 times higher in the presence of AF than in the absence of AF. Third, we demonstrate how distinct the statistical properties of pRRx are in SR and AF. Fourth, if HRV parameters are used to differentiate AF from SR, for example, in the machine learning algorithms, pRRx with x shorter than 50 should be applied.

In SR, the differences between the duration of successive RR intervals are much more limited than in AF. The RR interval for SR usually falls within 80 to 120% of the previous RR interval. For AF, no such limit exists. It results from a couple of physiological regulatory mechanisms controlling SR, which have weaker or no effect on the heart rate during AF.

SR originates in the sinus node, where pacemaker depolarization activity is regulated by several controlling mechanisms. The electrical depolarization from the sinus nodes travels across the right atrium and reaches the atrioventricular node. The atrioventricular conduction undergoes additional controlling regulations. During AF, thousands of cells from the border of pulmonary veins and the left atrium or both atria depolarize spontaneously in a relatively uncontrolled way. Atrial depolarizations reach the atrioventricular node in a more or less random order, which may or may not conduct them through the His system to both ventricles. The refractory period is probably the most important physiological mechanism that is still functioning; it may control the ventricle rate in AF. All cells of the atrioventricular node, His system, and ventricles have various refractory periods. During this period, cardiac cells below the atria cannot respond to electrical depolarization. The refractory period, particularly in the atrioventricular node, is under the strong control of the autonomic nervous system during SR. This control is less effective but is still present during AF. In other words, less controlling mechanisms over the ventricular rate in AF introduce some randomness to RR intervals and thus higher values of pRRx than in SR.

The limitations of the study must be recognized. It is an observational and retrospective study using ECG recordings from a single (Long-Term AF Database) database. To verify whether the results are not database-specific, we used optimal cutoffs of pRRx from LTAFDB and employed them to classify 60-s segments from MIT–BIH Atrial Fibrillation Database (250 Hz sampling frequency) [35,49]. The highest DOR (median 276) and accuracy (median 0.944) were obtained for pRR31 (in LTAFDB 194 and 0.928, respectively—the detailed results are shown in Appendix A). Moreover, these public databases have been used in several studies, and others can easily replicate our results. Other limitations are the arbitrary use of 60-ss ECG segments and the application of additional filters removing too short (<240 ms) or too long (>3000 ms) RR intervals to select ECG segments that might result in “too perfect” results. Thus, the interpretation of our findings must be limited only to the specific settings of the filters and ECG recording of 60-s duration.

Comparison of different methods and parameters is always complex and should never be based on a single approach or descriptor. As demonstrated, visual inspection is always essential—such a simple approach as the distribution analysis clearly shows how pRRx changes for various x, both for SR and AF. Unfortunately, presenting the actual data distribution is not a common approach. Next, correlation analysis is the most popular in many studies to present how well some parameters are correlated. Presented correlation heatmaps also show strong correlations for pRRx in a wide range of x values. Many studies stop at this point, without further and more detailed exploration. However, additional analyses with classic statistical methods, starting with differences analysis, followed by more advanced analyses such as ROC, the identification of cutoff points, and various classification measures, reveal huge differences between parameters that appear so well related. Summarizing this part, a set of several classical statistical methods for comparing various methods or parameters should be constantly employed in clinical medicine. Referring to one method, usually correlation analysis, can produce misleading conclusions and false diagnoses.

## 5. Conclusions

In conclusion, pRRx values for various x thresholds are not the same or interchangeable. Although most pRRx help detect AF, the parameters around pRR31 outperform others. If in a 60-s ECG the pRR31 is at least 68.79%, it is far more likely to be AF than SR. Using optimal pRRx instead of pRR50 should improve machine learning models for AF detection. Our results potentially apply to different biomedical systems used for screening for AF, e.g., long-term ECG Holter or bio-patch systems. However, we are aware that this potential should be validated using real-life ECG recordings acquired in clinical conditions, and it requires further prospective studies. It is worth noticing that the proposed approach for searching for the most valuable, from the point of view of AF detection, pRRx parameters can be repeated in other conditions and applied, for example, to specific ECG devices. Finally, pRR50 has been used in several studies as one of the HRV features incorporated in various machine learning models detecting AF. Replacing pRR50 with pRRx with a smaller x might improve the diagnostic properties of such models.

## Figures and Tables

**Figure A1:**
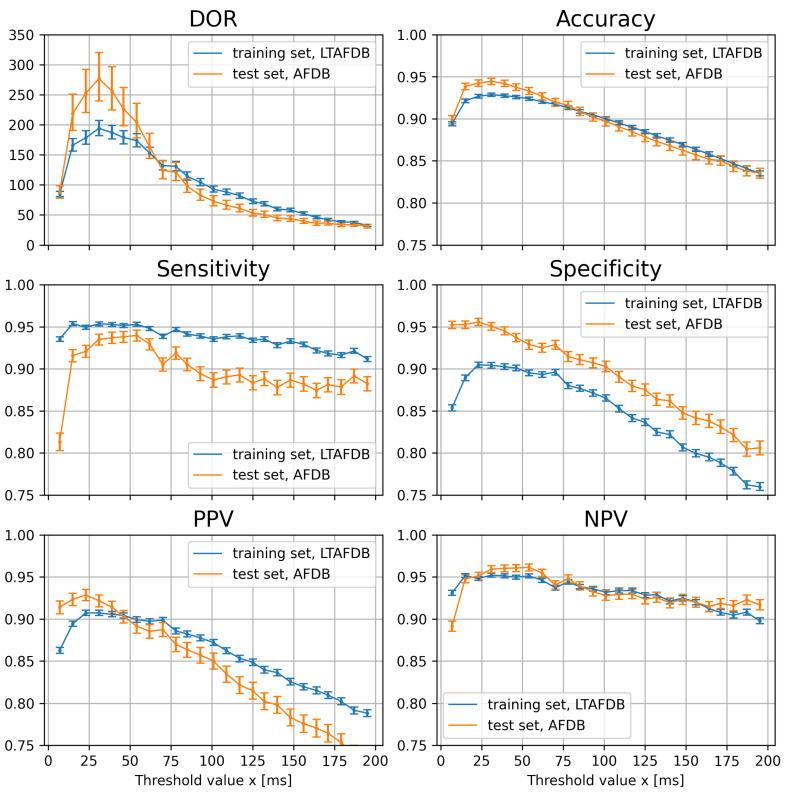
Medians and 95% confidence intervals of AF detection results in the training set Long-Term Atrial Fibrillation Database (LTAFDB) and the test set MIT–BIH Atrial Fibrillation Database (AFDB): diagnostic odds ratio (DOR, **top left**), accuracy (**top right**), sensitivity (**middle left**), specificity (**middle right**), positive predictive value (PPV, **bottom left**), and negative predictive value (NPV, **bottom right**).

**Figure A2:**
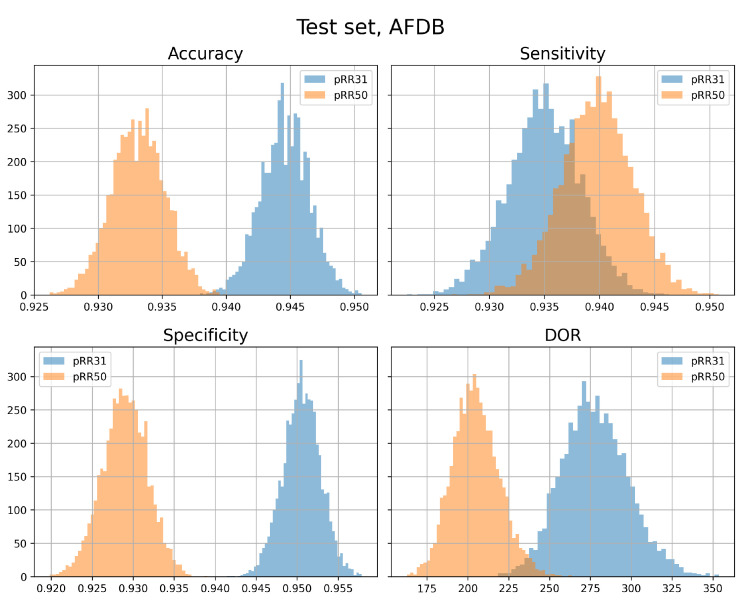
Histograms of atrial fibrillation detection results in the test set MIT–BIH Atrial Fibrillation Database (AFDB) for pRR31 and pRR50 (the percentages of successive RR intervals differing by at least 31 ms and 50 ms, respectively). DOR - diagnostic odds ratio.

## Data Availability

MIT–BIH Atrial Fibrillation Database (AFDB) [35,49], and Long-Term AF Database (LTAFDB) [35,36] were used in the study. They are available at https://physionet.org/content/afdb/1.0.0/ (accessed on 1 June 2022) and https://physionet.org/content/ltafdb/1.0.0/ (accessed on 1 June 2022), respectively.

## Appendix A. Results of Validation on MIT–BIH AF Database

We used the optimal cutoff values of pRRx parameters from the training set long-term Atrial Fibrillation Database (LTAFDB) to detect AF in the test set from the Massachusetts Institute of Technology–Beth Israel Hospital (MIT–BIH) Atrial Fibrillation Database (AFDB) [35,49]. Data preparation was the same as in the training set (Figure 1), and a nonparametric bootstrap with 5000 samples was used to estimate classification results’ distribution.

### Appendix A.1. Comparison between Training Set and Test Set Results

A comparison of the results of AF detection in the training set and the test set is presented in Figure A1. The classification metrics include the diagnostic odds ratio (DOR), accuracy, sensitivity, specificity, positive predictive value (PPV), and negative predictive value (NPV). In the test set, the highest DOR (median 276) and accuracy (median 0.944) are achieved for pRR31 (in training set 194 and 0.928, respectively). Peak values of DOR, accuracy, PPV, NPV, and specificity are higher in the test set AFDB, while sensitivity is higher in the training set LTAFDB in the whole range of threshold values x.

The difference between performance in the training set and the test set can be a result of one or more factors, including:

- Distinct patient populations in two databases:
  – LTAFDB: 84 subjects;
  – AFDB: 25 subjects.
- Various sampling frequencies:
  – LTAFDB: 128 Hz;
  – AFDB: 250 Hz.
- The different lengths of the ECG recording:
  – LTAFDB: 24 h (night and day);
  – AFDB: 10 h (unspecified time of day).
- Unequal proportions of 60-s segments with AF and SR:
  – LTAFDB: 32,769 SR, 32,141 AF (49.5% AF);
  – AFDB: 8836 SR, 5490 AF (38.3% AF).

### Appendix A.2. Comparison between pRR31 and pRR50

The histograms in Figure A2 show the distribution of four classification metrics (accuracy, sensitivity, specificity, and DOR) in AF detection in the test set using pRR31 and pRR50. For accuracy, specificity, and DOR, the results are higher for pRR31 than for pRR50. For sensitivity, there is an overlap between the distributions for pRR31 and pRR50, but the average is higher for pRR50.

Since not all the metrics had normal distribution (Shapiro–Wilk test) both for pRR31 and pRR50, we used the Wilcoxon test to compare the performance of pRR31 and pRR50. In all cases, the differences between the classification results cannot be explained by randomness.

### Appendix A.3. Conclusions from the Validation

The results presented in the Appendix A confirm the study’s findings. For AF detection in 60-s ECG, the most effective threshold value x in pRRx is not 50 ms (pRR50). Both in the training set LTAFDB and in the test set AFDB, pRR31 achieves the best performance, measured by accuracy and DOR. This phenomenon is not limited to a single ECG database; thus, pRR50 should not necessarily be a default pRRx parameter used in AF detection.
